# Proteinuria with extended VEGF inhibitor therapy in RAS-mutant metastatic colorectal cancer: incidence, interruptions, and exploratory survival analysis

**DOI:** 10.1007/s00384-026-05210-x

**Published:** 2026-08-04

**Authors:** Daichi Watanabe, Hironori Fujii, Yuto Miura, Misato Kani, Wakana Chikaishi, Jesse Yu Tajima, Akitaka Makiyama, Yunami Yamada, Koichi Ohata, Chiemi Hirose, Hirotoshi Iihara, Ryo Kobayashi, Nobuhisa Matsuhashi, Akio Suzuki

**Affiliations:** 1 Department of Pharmacy, Gifu University Hospital, Gifu, Japan; 2 Innovative and Clinical Research Promotion Center, Gifu University Hospital, Gifu, Japan; 3 Laboratory of Advanced Medical Pharmacy, Gifu Pharmaceutical University, Gifu, Japan; 4 Department of Surgical Oncology, Gifu University Graduate School of Medicine, Gifu, Japan; 5 Cancer Center, Gifu University Hospital, Gifu, Japan; 6 Patient Safety Division, Gifu University Hospital, Gifu, Japan; 7 Laboratory of Pharmacy Practice and Social Science, Gifu Pharmaceutical University, Gifu, Japan

**Keywords:** Proteinuria, Angiogenesis inhibitors, Colorectal neoplasms, Bevacizumab, Treatment outcome

## Abstract

**Purpose:**

Vascular endothelial growth factor inhibitors (VEGFIs) are essential for the treatment of metastatic colorectal cancer (mCRC). Given the ineffectiveness of epidermal growth factor receptor inhibitors in managing RAS-mutant mCRC, long-term VEGFI therapy is often required; however, it is associated with an increased risk of proteinuria. As the use of VEGFI in later-line mCRC treatment has become more prevalent, proteinuria has emerged as a critical dose-limiting toxicity. However, its effect on the treatment outcomes remains unclear. This study examined the incidence of proteinuria during extended VEGFI use and its effect on overall survival (OS) in RAS-mutant mCRC.

**Methods:**

This single-center retrospective study analyzed patients with RAS-mutant stage IV mCRC treated with first-line VEGFIs at Gifu University Hospital. Proteinuria incidence was estimated using the Kaplan–Meier method. OS from a 12-month landmark was compared according to the occurrence of dipstick proteinuria 2+ or greater within the first year using Cox regression.

**Results:**

Among 185 patients, the Kaplan–Meier cumulative incidence of dipstick proteinuria 2+ or greater was 11%, 37%, and 45% at 1, 2, and 3 years, respectively. Of 123 patients in the 12-month landmark cohort, 13 had early-onset proteinuria. The age- and sex-adjusted HR for subsequent mortality was 1.81 (95% CI 0.84–3.86; *p* = 0.128).

**Conclusions:**

Proteinuria accumulated during extended VEGFI therapy. Early-onset proteinuria may be associated with poorer subsequent OS, although the wide confidence interval precludes a definitive conclusion. These findings underscore the importance of careful proteinuria monitoring during long-term VEGFI treatment.

**Supplementary Information:**

The online version contains supplementary material available at https://doi.org/10.1007/s00384-026-05210-x.

## Introduction

Vascular endothelial growth factor inhibitors (VEGFIs) are critical agents for the treatment of metastatic colorectal cancer (mCRC). In first- and second-line therapies, VEGFIs such as bevacizumab (BEV), ramucirumab, and aflibercept have demonstrated improved efficacy when combined with oxaliplatin- and irinotecan-based regimens, as evidenced by several clinical trials [1–6]. Additionally, BEV allows continued treatment beyond disease progression, a strategy known as “Bevacizumab Beyond Progression.”[7] In later-line settings, adding BEV to trifluridine/tipiracil (TAS-102) significantly prolongs overall survival (OS) [8]. Similarly, regorafenib and fruquintinib, both of which exhibit VEGF receptor inhibitory activity, have also shown efficacy [9, 10]. Given their sustained therapeutic benefits, VEGFIs are widely used across the treatment continuum for mCRC, from first-line to late-line therapies.

Despite their efficacy, VEGFIs are associated with a dose-dependent increase in proteinuria [11], which poses significant clinical challenges. Proteinuria can progress to nephrotic syndrome [12] and is associated with an increased risk of kidney injury [13]. For bevacizumab, current guidance recommends withholding treatment when proteinuria reaches ≥ 2 g/24 h and resuming it once it decreases to < 2 g/24 h [14].

In RAS-mutated mCRC, in which epidermal growth factor receptor inhibitors are not viable options, VEGFIs serve as a cornerstone of long-term treatment. However, its prolonged use raises concerns regarding the management of proteinuria. A study of 46 ovarian cancer patients receiving extended VEGFI therapy reported that 49% developed proteinuria of any grade within a median follow-up period of 14 months [15]. Similarly, a study of multiple solid tumors found that 44.2% of patients receiving BEV developed proteinuria, leading to treatment discontinuation in 15.8% [16]. These findings underscore the challenges of managing proteinuria during extended VEGFI therapy, which can necessitate treatment cessation.

In mCRC, evidence of long-term VEGFI use remains scarce, particularly in later-line settings. Recent trials investigating TAS-102 with BEV and fruquintinib excluded patients with severe proteinuria (≥ 1 g/24 h or ≥ 2+ on qualitative tests) [8, 10], highlighting the potential impact of early-onset proteinuria on treatment continuation. However, the incidence of proteinuria during extended VEGFI use and its effects on treatment outcomes remain poorly understood.

Therefore, this study aimed to address two key challenges in long-term VEGFI therapy for mCRC: (1) quantifying the incidence of proteinuria during extended VEGFI use and (2) assessing the impact of early-onset proteinuria on overall treatment outcomes.

## Patients and methods

### Study design and setting

This single-center retrospective cohort study was conducted using electronic medical records from Gifu University Hospital. To evaluate proteinuria occurrence during long-term VEGFI use in mCRC, patients with RAS-mutant Stage IV mCRC were included. Two primary analyses were performed:

1. The incidence of proteinuria was estimated from VEGFI initiation to treatment discontinuation.
2. OS was compared between patients who developed urine dipstick proteinuria ≥ 2+ within 1 year and those who did not.

A 12-month threshold was selected to capture early proteinuria potentially associated with oxaliplatin- or irinotecan-based chemotherapy. This timeframe aligns with the median progression-free survival of 12.1 months reported in the TRIBE trial [3] and the median onset of proteinuria observed at 14 months [15]. Dipstick proteinuria 2+ or greater was selected for landmark classification because dipstick testing was repeated more frequently than UPCR in routine care and commonly served as a signal for quantitative evaluation or treatment review. The study was approved by the Ethics Committee of Gifu University Graduate School of Medicine (approval number 2023-286) and conducted in accordance with Japanese government guidelines for human research.

### Participants

Patients with RAS-mutant mCRC who began first-line VEGFI-containing chemotherapy at Gifu University Hospital between January 2010 and December 2023 were included in the study. Patients with baseline urine dipstick proteinuria ≥ 2+ were excluded.

### Variables and measurements

The baseline proteinuria status, patient demographics, and clinical covariates were recorded within 14 days of treatment initiation. The VEGFIs included BEV, ramucirumab, and aflibercept. Proteinuria was assessed using serial dipstick urinalysis and the urine protein-to-creatinine ratio (UPCR). Four proteinuria definitions were evaluated: dipstick 2+ or greater, a routinely available clinical signal that may prompt quantitative assessment or treatment review; dipstick 3+ or greater, a higher semiquantitative threshold; UPCR 2 or greater, a quantitative threshold for higher-level proteinuria; and a composite of dipstick 3+ or greater or UPCR 2 or greater, intended to capture higher-level proteinuria when UPCR testing was incomplete. Dipstick proteinuria 2+ or greater was used for landmark classification because dipstick testing was performed more consistently than UPCR testing in routine practice; this definition was not considered equivalent to quantitatively severe proteinuria.

### Statistical analysis

Descriptive statistics were used to summarize the data, with continuous variables presented as medians and interquartile ranges and categorical variables expressed as frequencies and percentages.

To evaluate cumulative proteinuria incidence, the time from the first VEGFI administration to proteinuria onset was measured using four criteria: a composite outcome of urine dipstick proteinuria 3+ or greater or UPCR 2 or greater (to account for incomplete UPCR measurements), UPCR 2 or greater, urine dipstick proteinuria 3+ or greater, and urine dipstick proteinuria 2+ or greater. Discontinuation of VEGFI treatment, hospital transfer, and conversion surgery were treated as censoring events. Cumulative incidence estimates with 95% confidence intervals (CIs) were calculated using the Kaplan–Meier method. As a sensitivity analysis, cumulative incidence functions were estimated using the Aalen–Johansen method, treating death and VEGFI discontinuation as competing events. VEGFI discontinuation was treated as a competing event only when at least 90 days of follow-up were available after the last VEGFI administration.

For OS analysis, a 12-month landmark was defined after treatment initiation. Patients alive and traceable at the landmark were included even if VEGFI treatment had already ended. For patients with less than 12 months of observation, exclusion reasons were classified hierarchically as death, salvage/conversion surgery, transfer or loss to follow-up, or other reasons. For Kaplan–Meier curves and Cox models, survival time started at the landmark and ended at death or censoring. Median follow-up from the landmark was estimated using the reverse Kaplan–Meier method. The primary Cox model adjusted for age and sex. Given the small number of patients in the early-onset group, additional sensitivity models separately added baseline creatinine clearance or treatment initiation year.

The proportion of VEGFI treatment interruptions, defined as cessation until proteinuria improved, was described across treatment cycles according to early-onset proteinuria status. Exploratory treatment-intensity analyses evaluated whether the last recorded VEGFI dose occurred before the 12-month landmark and calculated cumulative BEV-equivalent exposure. BEV exposure was expressed as administered mg/kg; ramucirumab and aflibercept were standardized per dose as (ramucirumab mg/kg/8) × 5 and (aflibercept mg/kg/4) × 5, respectively. This standardization reflects standard per-dose regimens and does not imply pharmacologic equivalence. Fixed-window exposure was accumulated through 365 days after the first VEGFI administration.

Factors associated with the onset of dipstick proteinuria (≥ 2 +) were analyzed using a univariable Cox proportional hazards model. Covariates included age (analyzed at three thresholds: < 65 vs. ≥ 65, < 70 vs. ≥ 70, and < 75 vs. ≥ 75 years), body mass index (BMI) (< 18.5, 18.5–25, or ≥ 25), creatinine clearance (< 60 vs. ≥ 60 mL/min, calculated by the Cockcroft-Gault formula), sex, baseline dipstick proteinuria, hypertension, and the use of antihypertensive medications.

Missing data were not imputed. As an exploratory study with no formal sample-size calculation, *p*-values were interpreted as indicators of statistical association rather than confirmatory hypothesis tests. All analyses were conducted using R version 4.4.1.

## Results

**Fig. 1:**
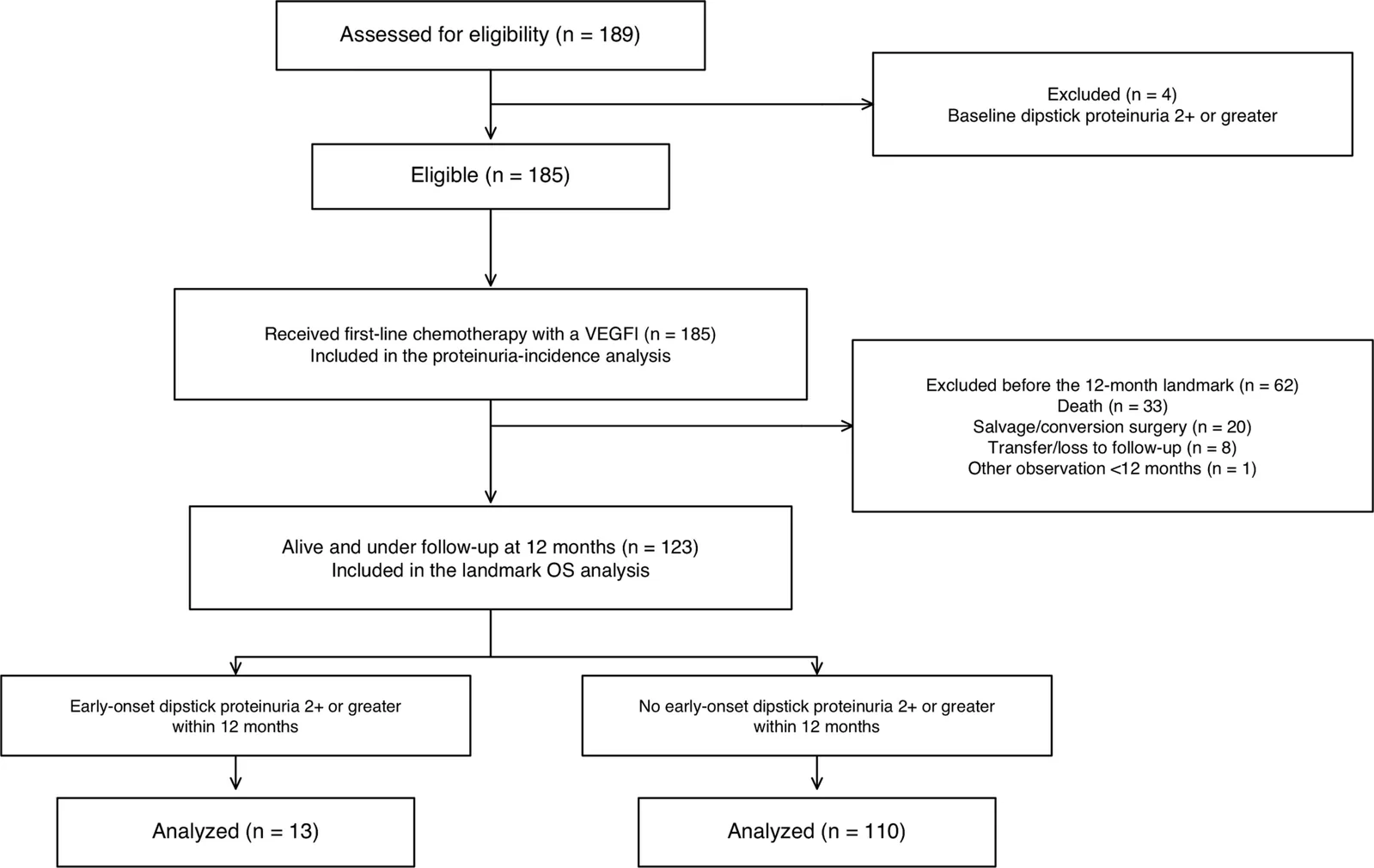
Patient flow diagram. A diagram illustrating the patient enrollment and exclusion process

The study design is illustrated in Online Resource 1, and patient flow is shown in Fig. 1. Of 189 patients who met the initial eligibility criteria, 4 with dipstick proteinuria 2+ or greater at treatment initiation were excluded, leaving 185 patients for the proteinuria-incidence analysis. Before the 12-month landmark, 62 patients were excluded: 33 died, 20 underwent salvage/conversion surgery, 8 were transferred or lost to follow-up, and 1 was administratively censored before reaching the landmark because treatment began less than 12 months before the data cutoff. The landmark cohort therefore included 123 patients: 13 with and 110 without dipstick proteinuria 2+ or greater within the first year. Patients who ended VEGFI treatment before 12 months remained eligible if they were alive and traceable at the landmark.

**Fig. 2:**
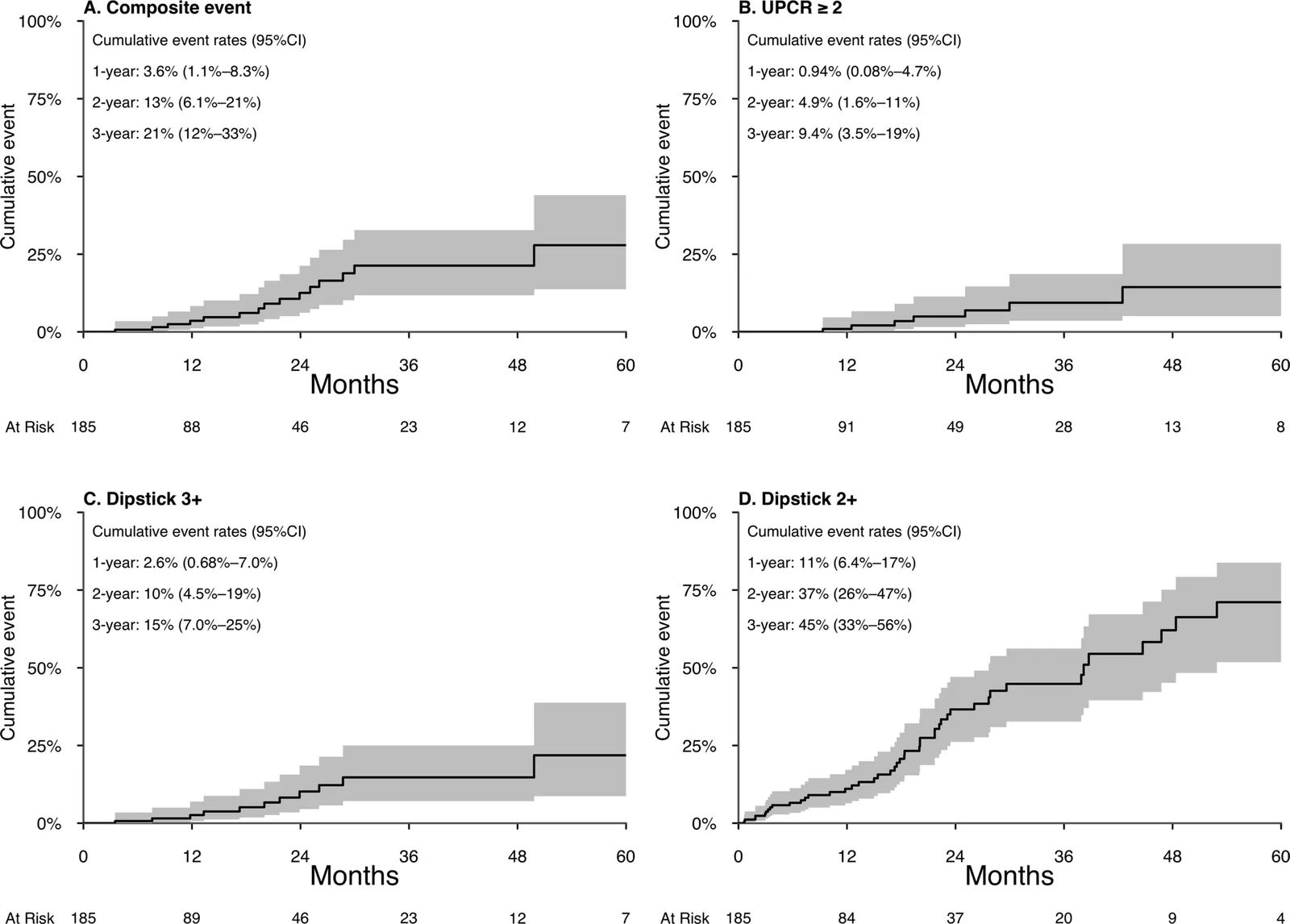
Cumulative incidence of proteinuria during anticancer treatment. Cumulative incidence rates with 95% confidence intervals are shown for different definitions of proteinuria: **A** composite outcome (UPCR ≥ 2 or dipstick ≥ 3+), **B** UPCR ≥ 2, **C** dipstick ≥ 3+, and **D** dipstick ≥ 2+

Patient characteristics at treatment initiation are summarized in Table 1. At baseline, seven patients had missing C-reactive protein (CRP) data, five had missing carcinoembryonic antigen (CEA) data, and eight had missing creatinine clearance data; all other variables were complete. The cohort comprised 122 patients (65.9%) aged 65 years or older and 95 male patients (51.4%). The distribution of the treatment initiation years was as follows: 22 patients (11.9%) from 2010 to 2013, 86 (46.5%) from 2014 to 2018, and 77 (41.6%) from 2019 to 2023. Online Resource 2 illustrates the changes in anticancer regimen usage across treatment cycles. Among patients who began treatment between 2010 and 2016, BEV combined with chemotherapy was the predominant regimen. Around the 20th treatment cycle, the use of BEV + TAS-102 increased notably, and this trend was even more pronounced among patients who initiated treatment between 2017 and 2023. No patient received fruquintinib. The median observation period in this study was 29.2 months (95% confidence interval [CI] 25.1–34.5). Figure 2 shows the cumulative incidence of proteinuria after treatment initiation. The 1-year and 3-year cumulative incidence rates were as follows: 3.6% (95% CI 1.1–8.3%) and 21% (95% CI 12–33%) for the composite outcome of urine dipstick proteinuria ≥ 3+ or UPCR ≥ 2; 0.94% (95% CI 0.08–4.7%) and 9.4% (95% CI 3.5–19%) for UPCR ≥ 2; 2.6% (95% CI 0.68–7.0%) and 15% (95% CI 7.0–25%) for urine dipstick proteinuria ≥ 3+; and 11% (95% CI 6.4–17%) and 45% (95% CI 33–56%) for urine dipstick proteinuria ≥ 2+. Among patients with dipstick proteinuria 2+ or greater, 27.7% did not undergo UPCR measurement.

**Table 1:** Baseline patient characteristics

Sample characteristics^a^	Overall
	***n*** = 185
Age, years (median [IQR])	69 (61–75)
< 65	63 (34.1%)
65–69	35 (18.9%)
70–74	38 (20.5%)
≥ 75	49 (26.5%)
Male	95 (51.4%)
BMI, kg/m^2^ (median [IQR])	22.1 (19.2–25.8)
< 18.5	32 (17.3%)
18.5–25	97 (52.4%)
≥ 25	56 (30.3%)
Creatinine clearance, mL/min (median [IQR])	76.3 (60.3–87.3)
< 30	1 (0.6%)
30–60	42 (23.7%)
≥ 60	134 (75.7%)
Albumin, g/dL (median [IQR])	4.0 (3.7–4.3)
AST, U/L (median [IQR])	21.0 (17.0–29.3)
ALT, U/L (median [IQR])	16.0 (11.0–23.0)
T-Bil, mg/dL (median [IQR])	0.6 (0.5–0.8)
CRP, mg/dL (median [IQR])	0.2 (0.1–0.6)
CEA, ng/mL (median [IQR])	13.9 (4.5–72.6)
Hypertension	64 (34.6%)
Antihypertensive medication	
None	125 (67.6%)
ARB	36 (19.5%)
Non-ARB	24 (13.0%)
Year of chemotherapy start	
2010–2013	22 (11.9%)
2014–2018	86 (46.5%)
2019–2023	77 (41.6%)

Abbreviations: *ALT* alanine aminotransferase, *ARB* angiotensin II receptor blocker, *AST* aspartate aminotransferase, *BMI* body mass index, *CEA* carcinoembryonic antigen, *CRP* C-reactive protein, *IQR* interquartile range *T-Bil* total bilirubin

^a^Reported as *n* (%) unless otherwise specified

In the competing-risk sensitivity analysis, the corresponding Aalen–Johansen cumulative incidence estimates at 1 and 3 years were 3.1% and 11.7% for the composite outcome, 0.6% and 5.1% for UPCR 2 or greater, 2.4% and 8.7% for dipstick proteinuria 3+ or greater, and 10.5% and 27.4% for dipstick proteinuria 2+ or greater, respectively (Online Resource 3).

**Fig. 3:**
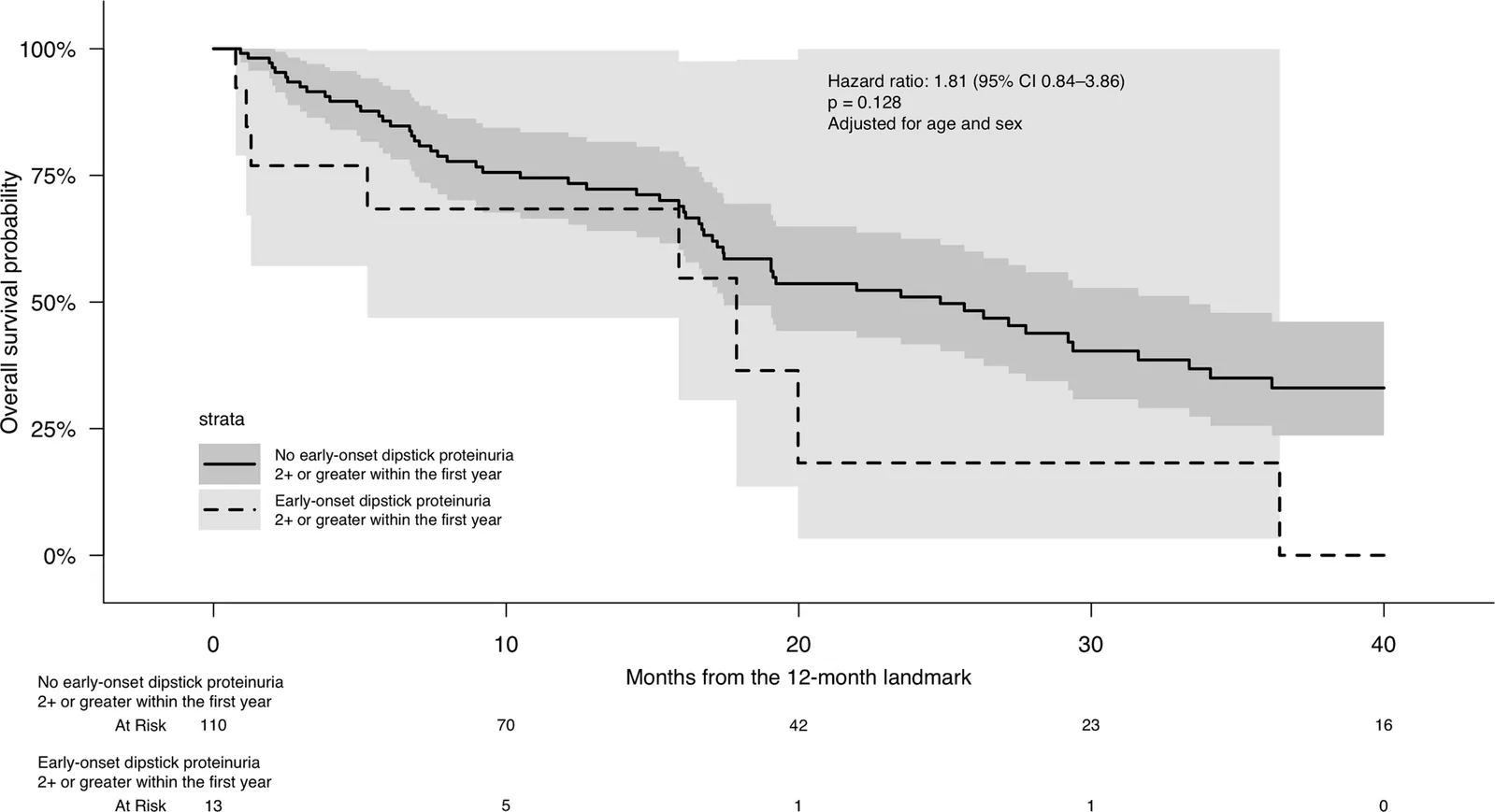
Landmark analysis of overall survival. Kaplan-Meier curves showing OS from the 12-month landmark, stratified by dipstick proteinuria 2+ or greater within the first year. Numbers at risk are shown from the landmark. The HR and *p*-value are from the age- and sex-adjusted Cox model

Figure 3 presents OS from the 12-month landmark. In the early-onset group, 8 of 13 patients died and 5 were censored; in the non-early-onset group, 67 of 110 patients died and 43 were censored. Across the entire landmark cohort, median follow-up from the landmark, estimated using the reverse Kaplan–Meier method, was 33.7 months (95% CI, 25.9–50.9). Median OS from the landmark was 18.3 months (95% CI, 5.5 to not estimable) in the early-onset group and 25.4 months (95% CI, 17.9–34.0) in the non-early-onset group. In the primary model adjusted for age and sex, the HR for mortality associated with early-onset proteinuria was 1.81 (95% CI, 0.84–3.86; *p* = 0.128). When creatinine clearance and treatment initiation year were added separately to the primary model, the HRs for early-onset proteinuria were 1.98 (95% CI, 0.91–4.29; *p* = 0.083; *n* = 118, 71 deaths) and 2.39 (95% CI, 1.06–5.40; *p* = 0.037; *n* = 123, 75 deaths), respectively (Table 2). Given the small number of patients in the early-onset group, the wide confidence intervals, and the variation in estimates across models, these findings should be interpreted as hypothesis-generating.

**Table 2:** Sensitivity analyses for early-onset proteinuria and OS after the 12-month landmark

Model	n	Deaths	HR (95% CI)	*p*-value
Early-onset proteinuria + age + sex	123	75	1.81 (0.84–3.86)	0.128
Basic model + creatinine clearance	118	71	1.98 (0.91–4.29)	0.083
Basic model + treatment initiation year	123	75	2.39 (1.06–5.40)	0.037

HRs compare early-onset with no early-onset proteinuria. The creatinine-clearance model used complete cases. The two additional covariates were entered in separate models because the exposed group was small

**Fig. 4:**
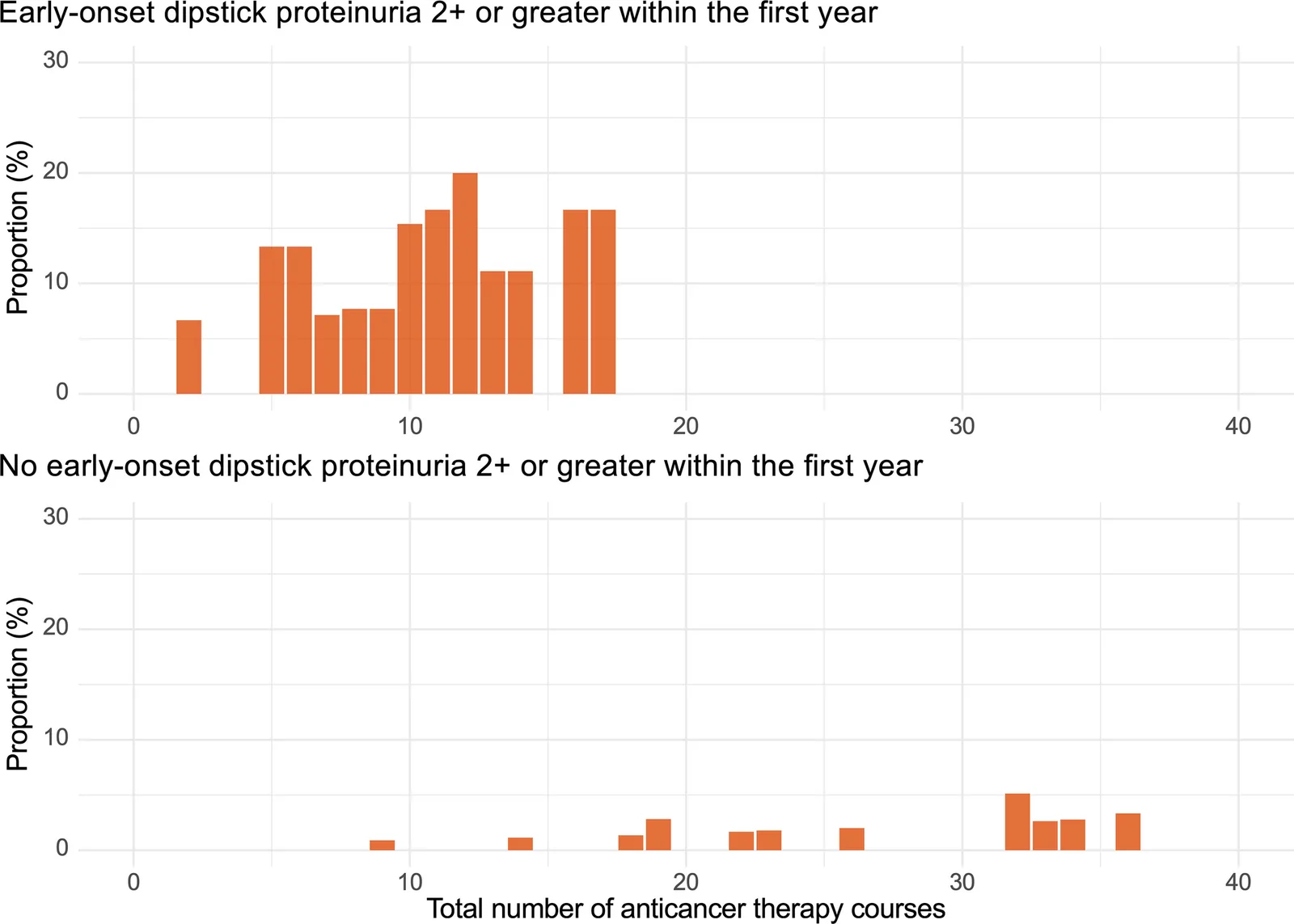
VEGF inhibitor interruption rates across treatment cycles. Interruption rates for each treatment cycle were calculated by dividing the number of patients who experienced a VEGF inhibitor interruption in that cycle by the total number of patients who received VEGF inhibitor treatment in the same cycle. These interruption rates are presented, stratified by the presence or absence of proteinuria (urine dipstick ≥ 2+) within the first year of treatment

In the descriptive cycle-specific analysis, VEGFI interruption rates were generally higher in the early-onset group than in the non-early-onset group (Fig. 4). To further characterize treatment delivery, the last recorded VEGFI administration was before the landmark in 6 of 13 patients (46.2%) in the early-onset group and 36 of 110 patients (32.7%) in the non-early-onset group. An exploratory Cox model adjusted for age and sex showed that last recorded VEGFI administration before the landmark was associated with poorer subsequent OS (HR 2.52, 95% CI 1.51–4.23; *p* < 0.001). Median cumulative BEV-equivalent exposure during the first 365 days after treatment initiation was 63.5 mg/kg (IQR, 57.2–76.3) in the early-onset group and 65.5 mg/kg (IQR, 36.3–90.8) in the non-early-onset group (*p* = 0.866).

Table 3 presents the results of univariate Cox regression analysis for the occurrence of dipstick proteinuria (≥ 2+). A higher age was correlated with an increased hazard of developing proteinuria, and statistical significance was observed using the 70-year cutoff (HR for ≥ 70 vs. < 70:1.97, 95% CI 1.07–3.63; *p* = 0.03). Though not statistically significant, elevated hazard ratios were also observed for age ≥ 65 years (HR 1.56; 95% CI, 0.84–2.88; *p* = 0.158) and ≥ 75 years (HR 1.49; 95% CI, 0.73–3.05; *p* = 0.27), as well as for creatinine clearance < 60 mL/min (HR 1.53; 95% CI, 0.73–3.21; *p* = 0.260). In contrast, no substantial associations were recognized for the other variables: male sex (HR 1.00; 95% CI, 0.56–1.78; *p* = 0.993), body mass index (HR 0.73; 95% CI, 0.31–1.70; *p* = 0.5) for < 18.5 vs. 18.5–25 and HR 1.08 [95% CI, 0.56–2.08; *p* = 0.8] for ≥ 25 vs. 18.5–25, baseline dipstick proteinuria of 1+ versus < 1+ (HR 0.59; 95% CI, 0.08–4.34; *p* = 0.603), hypertension (HR 0.85; 95% CI, 0.55–1.31; *p* = 0.462), or antihypertensive medications (HR 1.03 [95% CI, 0.45–2.35; *p* = 0.945] for angiotensin receptor blocker (ARB) treatment vs. no treatment and HR 1.36 [95% CI, 0.60–3.11; *p* = 0.466] for non-ARB medications vs. no treatment).

**Table 3:** Univariable Cox regression for dipstick proteinuria ≥ 2+

Variable^a^	HR (95% CI)	*p*-value
Age^b^		
≥ 65 (reference is < 65)	1.56 (0.84–2.88)	0.158
≥ 70 (reference is < 70)	1.97 (1.07–3.63)	0.03
≥ 75 (reference is < 75)	1.49 (0.73–3.05)	0.27
Sex		
Female (reference)	-	-
Male	1.00 (0.56–1.78)	0.993
Body mass index		
18.5–25 (reference)	-	-
< 18.5	0.73 (0.31–1.70)	0.5
≥ 25	1.08 (0.56–2.08)	0.8
Creatinine clearance (mL/min)		
≥ 60 (reference)	-	-
< 60	1.53 (0.73–3.21)	0.260
Baseline proteinuria on dipstick urinalysis		
< 1+ (reference)	-	-
1+	0.59 (0.08–4.34)	0.603
Hypertension		
No (reference)	-	-
Yes	0.85 (0.55–1.31)	0.462
Antihypertensive medication		
None (reference)	-	-
ARB treatment	1.03 (0.45–2.35)	0.945
Non-ARB antihypertensive medication	1.36 (0.60–3.11)	0.466

Abbreviations: *ARB* angiotensin receptor blocker, *HR* hazard ratio

^a^All factors were added to the Cox regression model as single explanatory variables and all factors use information at treatment initiation

^b^Age was analyzed using three separate dichotomous cutoffs (≥ 65 vs < 65, ≥ 70 vs < 70, ≥ 75 vs < 75) in independent univariable models

## Discussion

This study investigated proteinuria during long-term VEGFI use in RAS-mutant mCRC and explored its association with subsequent survival when it occurred early. The adjusted point estimate was consistent with a higher mortality hazard (HR 1.81), but the 95% CI was wide (0.84–3.86), included no association, and the *p*-value was 0.128. The finding should therefore be interpreted as a possible association and a hypothesis-generating signal rather than evidence that early-onset proteinuria worsens prognosis.

Notably, this is the first study to investigate the long-term profile of proteinuria in patients with mCRC from first-line through later-line treatments. As most patients received VEGFIs throughout their anticancer treatment, this enabled a thorough evaluation of proteinuria during extended VEGFI exposure. The median observation period was 29.2 months, which is substantially longer than prior studies on proteinuria occurrence in other cancer types [15, 16].

The cumulative incidence of proteinuria increased with treatment duration across the evaluated definitions, including clinically stronger endpoints based on dipstick 3+ or UPCR 2 or greater. This accumulation may have practical implications for the continuation or selection of later-line antiangiogenic treatments, such as BEV plus TAS-102 or fruquintinib, and underscores the importance of proteinuria monitoring and management throughout the treatment course.

In the primary 12-month landmark analysis, early-onset dipstick proteinuria showed a trend toward poorer subsequent OS, although the wide confidence interval precluded a definitive conclusion. In exploratory treatment-delivery analyses, last recorded VEGFI administration before the landmark was descriptively more frequent in the early-onset group, and this proxy was associated with poorer subsequent OS (age- and sex-adjusted HR 2.52, 95% CI 1.51–4.23). In contrast, fixed-window cumulative BEV-equivalent exposure was similar between the early- and non-early-onset groups. These findings highlight the need for further investigation of the relationships among early proteinuria, the timing of VEGFI cessation, treatment exposure, and subsequent survival.

Related studies of advanced hepatocellular carcinoma treated with atezolizumab plus BEV found that early BEV interruption was associated with a lower objective response rate and poorer PFS and OS [17], whereas proteinuria was associated with improved outcomes [18, 19]. In advanced breast cancer, early proteinuria and hypertension during BEV-paclitaxel treatment were associated with a stronger antitumor response but not improved PFS [20]. Mechanisms remain unclear, and current evidence is insufficient to establish proteinuria as a biomarker for anticancer activity.

In this study, older age was associated with an increased incidence of dipstick proteinuria (≥ 2+), with statistical significance observed using the 70-year cutoff (HR, 1.97; 95% CI, 1.07–3.63; *p* = 0.03). Lower creatinine clearance (< 60 mL/min vs ≥ 60 mL/min) was also associated with an increased incidence (HR 1.53; 95% CI, 0.73–3.21; *p* = 0.260). Therefore, these findings suggest that older age, potentially along with an associated decline in renal function, could be considered a risk factor for the development of proteinuria. As impaired renal function is an established risk factor, as supported by previous reports [21], careful monitoring is essential, particularly for older patients and those with reduced renal function.

### Potential limitations

This study has several limitations. First, it was a single-center retrospective cohort study. Second, dipstick results can be influenced by urine concentration, hydration status, infection, and hematuria. Moreover, 27.7% of patients with dipstick proteinuria 2+ or greater did not undergo UPCR measurement, potentially leading to exposure misclassification. Third, only 13 patients in the landmark cohort had early-onset proteinuria. The resulting wide confidence intervals, limited statistical power, and model-dependent estimates preclude definitive conclusions. Important prognostic factors could not be included in the models, and unmeasured and residual confounding remain likely. Fourth, because VEGFI discontinuation was determined retrospectively from treatment records using an operational definition rather than prospectively adjudicated, the competing-risk estimates may be sensitive to this definition and subject to misclassification. Fifth, the prognostic significance of more severe early proteinuria, defined by dipstick 3+ or UPCR 2 or greater, could not be evaluated because of the small number of events. Sixth, no patient received fruquintinib; therefore, the implications for treatment with this agent are indirect and require further evaluation. Finally, the standard BEV dose in this cohort was 5 mg/kg every 2 weeks, which is lower than some regimens used for other cancer types. Generalization of these findings across cancer types and VEGFI dosing regimens therefore requires caution.

## Conclusions

In conclusion, proteinuria accumulated to a clinically relevant extent during long-term VEGFI treatment in patients with RAS-mutant mCRC. Early-onset dipstick proteinuria showed a trend toward poorer subsequent OS, although the wide confidence interval precluded a definitive conclusion. These findings underscore the importance of careful proteinuria monitoring and management throughout extended VEGFI therapy[JM5].

## Supplementary Information

Below is the link to the electronic supplementary material.

ESM 1 (PDF 1.23 MB)

## Data Availability

No datasets were generated or analysed during the current study.
