# Supplementary material for: Proteinuria with extended VEGF inhibitor therapy in RAS-mutant metastatic colorectal cancer: incidence, interruptions, and exploratory survival analysis
Source: Int J Colorectal Dis. 2026 Aug 4;41(1):175. doi: 10.1007/s00384-026-05210-x (PMC13719852; doi:10.1007/s00384-026-05210-x)
Supplement: Supplementary file 1 [file 384_2026_5210_MOESM1_ESM.pdf]

# Proteinuria With Extended VEGF Inhibitor Therapy in RAS-Mutant Metastatic Colorectal Cancer: Incidence, Interruptions, and Exploratory Survival Analysis

Author Names: Daichi Watanabe[1,2], Hironori Fujii[1], Yuto Miura[3], Misato Kani[3], Wakana Chikaishi[4], Jesse Yu Tajima[4], Akitaka Makiyama[5], Yunami Yamada[1], Koichi Ohata[1], Chiemi Hirose[1], Hirotoshi Iihara[1,6,7], Ryo Kobayashi[1,3], Nobuhisa Matsuhashi[4], Akio Suzuki[1,3]

**Journal Name:** International Journal of Colorectal Disease

**Affiliations:**

[1] Department of Pharmacy, Gifu University Hospital, Gifu, Japan.

[2] Innovative and Clinical Research Promotion Center, Gifu University Hospital, Gifu, Japan.

[3] Laboratory of Advanced Medical Pharmacy, Gifu Pharmaceutical University, Gifu, Japan.

[4] Department of Surgical Oncology, Gifu University Graduate School of Medicine, Gifu, Japan

5. Cancer Center, Gifu University Hospital, Gifu, Japan.

6. Patient Safety Division, Gifu University Hospital, Gifu, Japan.

7. Laboratory of Pharmacy Practice and Social Science, Gifu Pharmaceutical University, Gifu, Japan.

**Corresponding Author:**

Dr. Hironori Fujii

Department of Pharmacy

Gifu University Hospital, Gifu, Japan.

Email: fujii.hironori.u5@f.gifu-u.ac.jp

**Supplementary Information**

**Online Resource 1 Study design diagram.**

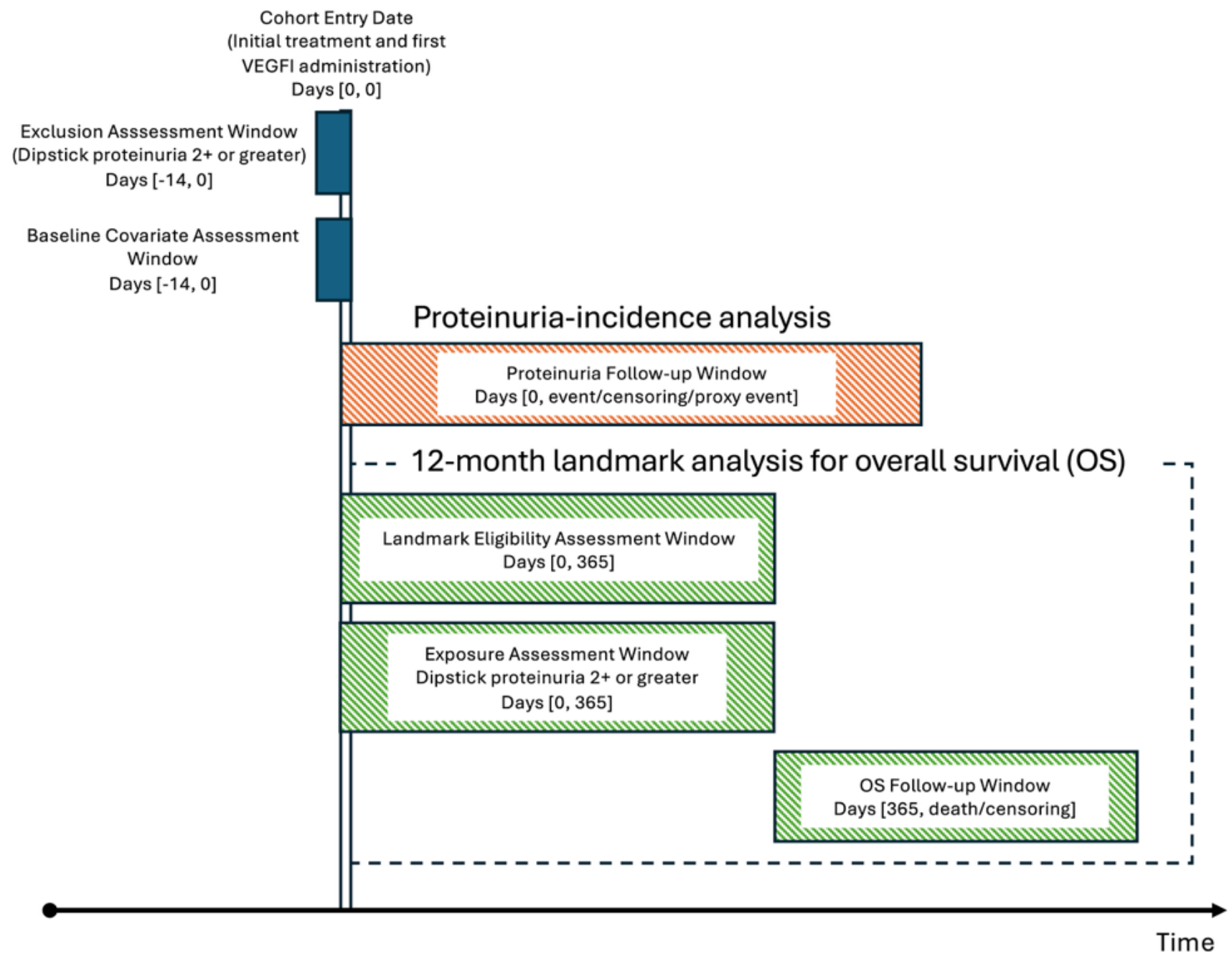


A diagram illustrating the study design. Proteinuria follow-up began at the first VEGFI administration. In the primary Kaplan–Meier analysis, VEGFI discontinuation, hospital transfer, and conversion surgery were censoring events; in the competing-risk sensitivity analysis, death and VEGFI discontinuation were treated as competing events. For the OS analysis, patients alive and traceable at the 12-month landmark were included even if VEGFI treatment had ended earlier. Patients who died, underwent salvage/conversion surgery, were transferred or lost to follow-up, or began treatment less than 12 months before the administrative data cutoff were excluded. Early-onset proteinuria was defined as dipstick proteinuria 2+ or greater within the first 365 days, and OS was measured from the landmark to death or censoring.

Abbreviations: OS, overall survival; VEGFI, vascular endothelial growth factor inhibitor.

**Online Resource 2 Changes in anti-cancer regimen usage across treatment cycles.**

(a)

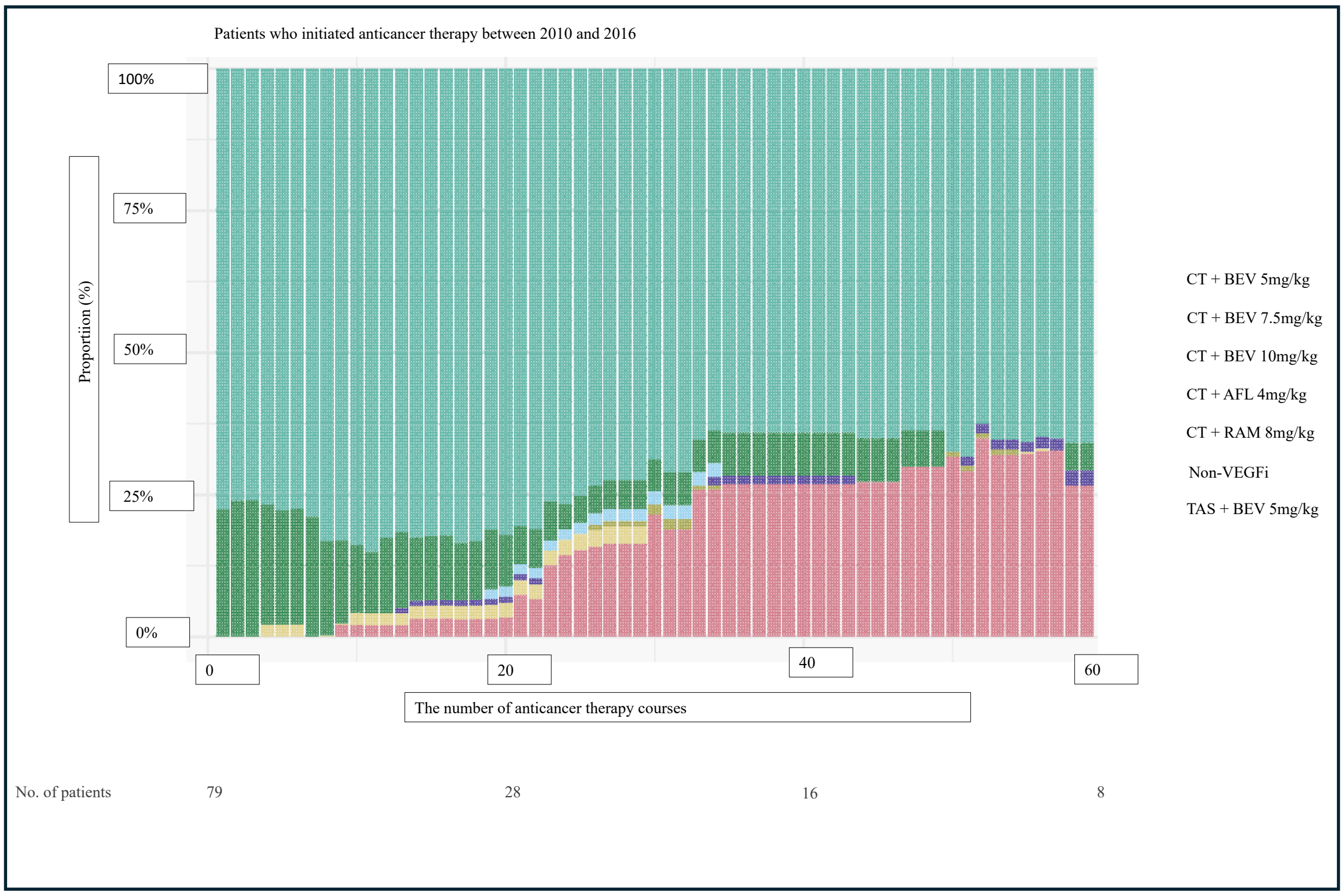


(b)

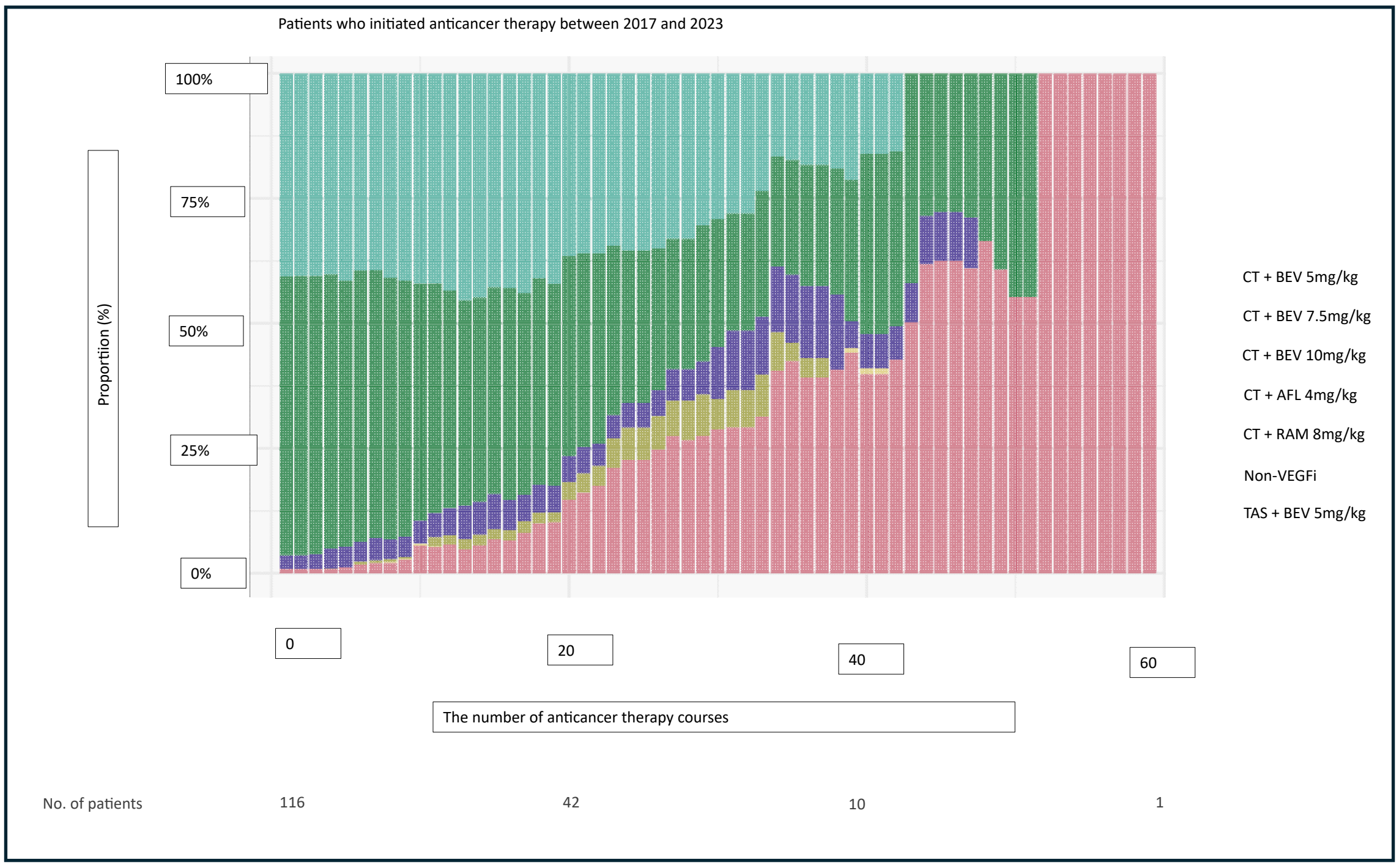


The proportion of each regimen used in each treatment cycle. This proportion was calculated by

dividing the number of patients who received each regimen in a given cycle by the total number of patients who received anticancer treatment in that same cycle. (a) Data for patients who initiated first-line treatment between 2010 and 2016. (b) Data for patients who initiated first-line treatment between 2017 and 2023.

**Online Resource 3 Competing-risk estimates of cumulative proteinuria incidence.**

The Aalen–Johansen sensitivity analysis treated death and VEGFI discontinuation as competing events. VEGFI discontinuation was treated as a competing event only when at least 90 days of follow-up were available after the last administration.

| Proteinuria definition | Month | Proteinuria CIF (95% CI), % |
|---|---|---|
| Dipstick 2+ or greater | 12 | 10.5 (6.4–15.6) |
| Dipstick 2+ or greater | 24 | 24.1 (17.6–31.2) |
| Dipstick 2+ or greater | 36 | 27.4 (20.4–34.8) |
| Dipstick 3+ or greater | 12 | 2.4 (0.8–5.7) |
| Dipstick 3+ or greater | 24 | 6.1 (3.0–10.9) |
| Dipstick 3+ or greater | 36 | 8.7 (4.7–14.3) |
| UPCR 2 or greater | 12 | 0.6 (0.1–3.2) |
| UPCR 2 or greater | 24 | 3.5 (1.3–7.4) |
| UPCR 2 or greater | 36 | 5.1 (2.3–9.8) |
| Dipstick 3+ or greater or UPCR 2 or greater | 12 | 3.1 (1.1–6.6) |
| Dipstick 3+ or greater or UPCR 2 or greater | 24 | 7.5 (4.0–12.5) |
| Dipstick 3+ or greater or UPCR 2 or greater | 36 | 11.7 (7.0–17.8) |

Values are estimates (95% CIs). Pointwise 95% CIs for the CIFs were calculated from the standard errors of the Aalen–Johansen estimator. CIF, cumulative incidence function; UPCR, urine protein-to-creatinine ratio; VEGFI, vascular endothelial growth factor inhibitor. Death and VEGFI discontinuation were treated as competing events; VEGFI discontinuation was assessed only when at least 90 days of follow-up were available after the last administration.
